# Vaginal Urinary Calculi Formation Secondary to Vaginal Mesh Exposure with Urinary Incontinence

**DOI:** 10.1155/2017/8710315

**Published:** 2017-08-22

**Authors:** Kara M. Griffiths, Geoffrey D. Towers, Jerome L. Yaklic

**Affiliations:** Obstetrics and Gynecology, Wright State University, Boonshoft School of Medicine, Dayton, OH, USA

## Abstract

**Background:**

Vaginal stones may form in the setting of mesh exposure with urinary incontinence. This report serves to help understand the presentation, evaluation, and management of vaginal urinary stones.

**Case:**

A 68-year-old female presented with a vaginal calculus. She had a history of anterior and posterior polypropylene mesh placement for prolapse 7 years earlier and urinary incontinence. The stone was identified on a portion of exposed mesh and removed in office. Pathology confirmed urinary etiology. The exposed mesh resolved with topical estrogen. Cystourethroscopy excluded urinary fistula and bladder mesh erosion.

**Conclusions:**

When identified, a vaginal calculus should be removed and evaluated for composition. Cystourethroscopy should be performed to assess potential urinary tract fistulas and mesh erosion. Additional imaging should be considered.

## 1. Introduction

Vaginal stones are uncommon [1]. They can be primary, resulting from urinary stasis in the vagina, or secondary, forming due to a present nidus [2]. This case involved stone formation in the vaginal fourchette as a result of vaginal prolapse mesh exposure in the setting of urinary incontinence. Since 2004, the use of vaginal mesh has greatly increased in the treatment of pelvic organ prolapse and urinary incontinence. Although patient and provider preference has trended away from transvaginal mesh for pelvic prolapse since the advent of the FDA mesh warning, mesh continues to be used in many instances following the joint recommendations of the American College of Obstetricians and Gynecologists and the American Urogynecologic Society [3, 4]. Furthermore, polypropylene mesh midurethral slings are now the “standard of care” in surgical treatment for stress urinary incontinence [5]. With a vaginal mesh exposure rate of 5–19% and midurethral sling erosion rate of <5%, the formation of vaginal stones in the setting of exposure and incontinence may become more common [6].

## 2. Case Presentation

**Figure 1:**
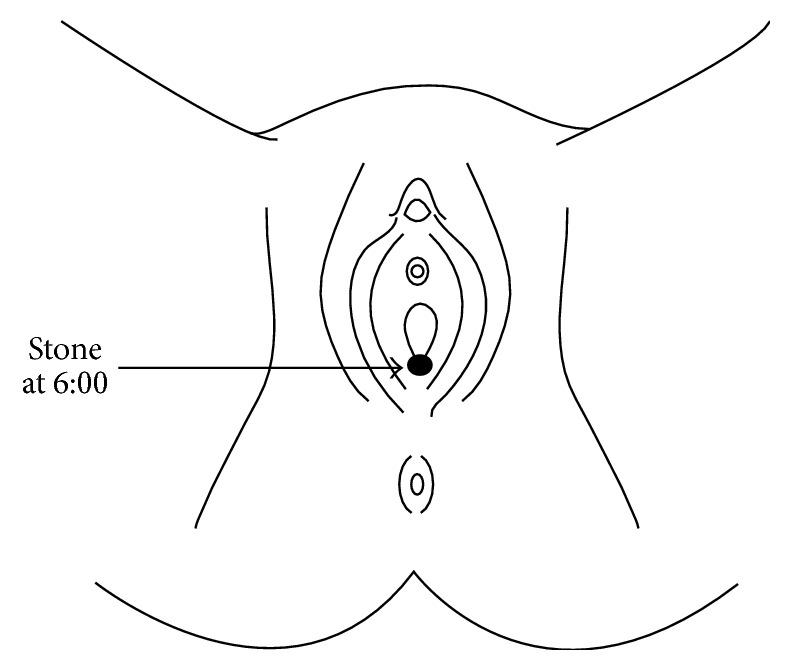
Diagram of the 5 × 8 mm stone at the vaginal fourchette.

This is a case of a 68-year-old female with a history of pelvic organ prolapse previously treated with an anterior and posterior polypropylene mesh (Prolift, Ethicon) seven years earlier who presented for evaluation of suspected vaginal mesh exposure. The patient's medical history is significant for three prior vaginal births and significant mixed urinary incontinence for which she wears a pad daily. Her presenting symptom was that she felt a “firm string,” which she initially thought was due to either mesh exposure or a retained portion of a Poise Impressa device. She denied any pain or vaginal bleeding. On physical exam, she was noted to have apical vaginal prolapse 2-3 cm proximal to the hymen with a lax perineum, and a stone measuring approximately 5 × 8 mm was present at the vaginal fourchette (Figure 1). The stone was attached to a 2 × 2 mm area of exposed vaginal mesh just superior to the hymenal remnant. There was no visible evidence of a fistula. The stone was easily removed in office during examination with pathology confirming a weight of 121 mg and a 70% struvite and 30% dahllite composition. The area of mesh exposure was treated with vaginal topical estrogen cream for four weeks, and the erosion completely resolved with no visible or palpable mesh exposure. Cystourethroscopy excluded mesh erosion into the bladder or the presence of a fistula.

## 3. Discussion

Urinary calculi forming outside the urinary tract are very rare [1]. We searched PubMed using the terms “mesh exposure,” “vaginal stone,” and “urinary incontinence” without any limitations and found only one other case of vaginal calculi formation resulting specifically from vaginal mesh exposure. We were unable to identify any cases with stone formation in the posterior vagina. The primary etiology of vaginal stones is via a vesicovaginal fistula in the setting of a urinary tract infection with the majority of these stones being struvite stones [7]. The second most common cause of vaginal urinary stones is the presence of a foreign body in the vagina [2].

Vaginal stones are considered either primary or secondary. Primary stones are thought to occur from urinary stasis in the vagina, whereas secondary stones form as urine crystalizes around a foreign body in the vagina [2]. Reports of primary stones have occurred with urethrovaginal fistulas forming after trauma in association with vaginal stenosis, urinary incontinence secondary to neurologic disorders, ectopic ureters, and urethral diverticula [2, 8, 9]. Secondary stones have been reported primarily in cases of vesicovaginal fistulae with cases involving long-term pessary use [10], an embedded displaced intrauterine contraceptive device [11], and retained surgical gauze [12, 13]. A report of vaginal stone formation on eroded tension free vaginal tape mesh is also documented [14]. Only one case was found similar to ours involving a woman with mixed urinary incontinence and vaginal mesh exposure, which was treated with transvaginal stone removal [15].

The presence of a vaginal stone should be considered if a patient presents with vaginal pain/discomfort, dyspareunia, partner pain with intercourse, dysuria, palpation of mesh/foreign body, or vaginal bleeding or discharge [10, 11, 14, 16, 17]. If unable to diagnose a vaginal stone on exam, X-ray imaging should be considered [8]. The etiology of the urinary salts forming these stones should also be explored via either intravenous pyelography, cystourethroscopy, CT, or MRI to evaluate for ectopic ureter, urethrovaginal or vesicovaginal fistula, or urethral diverticulum [16].

The treatment of vaginal stones secondary to mesh exposure is typically achieved via vaginal excision with removal of the stone and exposed mesh. In cases of large vaginal calculi, the use of lithotripsy or partial morcellation with episiotomy has been described [9].

Our patient's presenting symptom was that she felt the stone thinking it might be exposed mesh. Unlike most patients, she did not present with pain, urinary complaints, or vaginal bleeding. Her stone was easily removed in office and likely formed due to persistent exposure of the mesh to a urine-soaked pad due to her urinary incontinence. A urologic fistula was excluded with cystourethroscopy. Due to the increased use of vaginal prolapse mesh and midurethral slings, we anticipate vaginal stone formation to be a more prevalent complication, and we hope this report of our patient will aid in the future recognition and evaluation of vaginal stones.
